# Community‐Based Projects for Energy Transition: Citizen Profiling Through Psychological and Social Drivers

**DOI:** 10.1002/jcop.70131

**Published:** 2026-07-13

**Authors:** Evelyn De Simone, Alessia Rochira, Fortuna Procentese, Flora Gatti, Biagio Marano, Francesca D'Errico, Rosa Scardigno, Carmela Sportelli, Terri Mannarini

**Affiliations:** ^1^ Department of Human and Social Sciences University of Salento Lecce Italy; ^2^ Department of Humanities University of Naples Federico II Napoli Italy; ^3^ Department of Social Sciences University of Naples Federico II Napoli Italy; ^4^ Department of Surgery, Medicine, Dentistry, and Morphological Sciences University of Modena and Reggio Emilia Reggio Emilia Italy; ^5^ Department of Educational Sciences, Psychology, Communication University of Bari Bari Italy

**Keywords:** community participation, ecological systems theory, Italy, latent class analysis, motivation, renewable energy, social values

## Abstract

This study investigated psychosocial profiles of potential renewable energy community (REC) participants in Italy, examining how individual‐level, community‐level, and societal‐level factors combine to shape participation intentions. Latent Profile Analysis identified distinct profiles among Italian citizens (*N* = 580) using 11 indicators: Pro‐environmental Values, Civic Engagement, Intrinsic/Extrinsic Motivation, RECs acceptability (individual level); Sense of Community, Institutional Trust, Common Good Orientation, Sense of Responsible Togetherness (community level); and communication clarity (societal level). Three distinct profiles emerged: *Civic‐minded Interested* (35.5%), *Uncommitted* (56.4%), and *Private‐minded Uninterested* (8.1%). Counterintuitively, the Private‐minded profile showed the highest stated participation intention (72.3%), followed by Civic‐minded (67.0%) and Uncommitted (60.2%), though this overall pattern was non‐significant (*p* = 0.110). No significant differences emerged across profiles in preferred level of engagement. Findings reveal the multilevel, ecological nature of REC participation while highlighting the distinction between stated intentions and the psychosocial resources that typically sustain actual engagement. Results underscore the need for differentiated engagement strategies and community capacity building tailored to distinct psychosocial configurations.

## 1 Introduction

Renewable energy communities (RECs) represent collective entities where citizens, social entrepreneurs, and public authorities participate in clean energy production and consumption, emphasizing decentralization and citizen involvement (Soeiro and Ferreira Dias 2020a). Within RECs, members can engage in different roles. *Consumers* are members who join RECs primarily to consume shared renewable energy generated by the community's installations. *Prosumers*, by contrast, take on a dual role: They both produce renewable energy through their own installations (such as rooftop solar panels) and consume energy from the community system (De Simone et al. 2025).

RECs represent a transformative approach to energy transition, fundamentally reimagining the relationship between citizens and energy systems. They are not merely technical infrastructures, but social innovation processes that both depend upon and contribute to community development. RECs position community members as active participants in collectively producing, consuming, and managing renewable energy (Soeiro and Ferreira Dias 2020b; Wittmayer et al. 2021). This shift from passive consumption to active participation reflects broader principles of energy democracy, aiming to redistribute power and benefits within the energy sector while promoting social equity and community ownership (Allen et al. 2019). Within this collective framework, individual members may engage in different roles—as consumers who primarily use shared renewable energy, or as prosumers who also produce energy through their own installations.

Despite their value, the development and maintenance of RECs face significant challenges that extend beyond technical and economic considerations.

In particular, citizens' engagement in RECs projects represents one of the most remarkable issue that has attracted the interest of scholars and practitioners (Goedkoop et al. 2022; Horstink et al. 2020; Brambati et al. 2022; Conradie et al. 2021; Morgan and Canfield 2021; Soeiro and Ferreira Dias 2020a; Cabarcos et al. 2020; Rogers et al. 2008; Lupi et al. 2021; Soeiro and Ferreira Dias 2020b; Lizarralde et al. 2021; Young and Brans 2017). In this regard, recent evidence highlights that citizens may exhibit substantial heterogeneity in their potential participation in RECs (De Simone et al. 2025) and that such a heterogeneity might have important practical implications for the successful development of REC projects (Spurk et al. 2020). While participation trajectories involve multiple stages from initial interest to sustained engagement, understanding the psychosocial factors shaping citizens' willingness to participate represents a crucial foundation for these pathways. However, traditional “one‐size‐fits‐all” approaches to community engagement might be highly ineffective in capturing the complex ways in which motivations, attitudes, and contextual factors combine within individuals (Bergman & Trost 2006; Morin and Marsh 2015; Lizarralde et al. 2021; Lupi et al. 2021). Indeed, there is a need for a framework capable of identifying qualitatively different subgroups based on how multiple characteristics cluster together, an approach that shifts the unit of analysis from variables to persons (Howard and Hoffman 2018; Spurk et al. 2020).

This necessity motivates the present study, which adopts a person‐centered approach to identify distinct ways in which individual, community‐level, and societal‐level factors combine within community members to shape their potential participation. The person‐centered perspective, which guided this study, afforded both theoretical and practical advantages. By doing so, we intended to shed light on the multidimensional nature of citizens' decision‐making processes while enabling the development of differentiated engagement strategies tailored to specific population segments (Howard and Hoffman 2018; Morin and Marsh 2015).

## 2 Theoretical Framework

Citizens' participation in RECs is a relatively novel subject of investigation within social and community psychology. Drawing on ecological systems theory (Bronfenbrenner 1989), REC participation may be understood as embedded within nested systems of influence. This multilevel perspective recognizes that citizens' decisions to engage in RECs emerge from the interplay between their personal values and motivations (individual level), the social and institutional resources available in their communities (community level), and the broader policy and informational environment (societal level).

Although multiple factors shape REC development—including regulatory frameworks and financial incentives (Broska et al. 2022; Gregg et al. 2020), the presence of local champions and trusted initiators (Süsser et al. 2017; Young and Brans 2017), and technological considerations (Cabarcos et al. 2020)—a recent systematic review (De Simone et al. 2025) reveals that psychosocial factors consistently emerge as critical determinants of citizens' initial willingness to participate. Building on this evidence, the present study focuses on 11 psychosocial dimensions that (a) consistently emerged as relevant across diverse European contexts in our review, (b) represent theoretically distinct yet interrelated aspects of collective action, and (c) can be assessed among potential participants prior to actual REC membership. This selection spans individual orientations and motivations, community‐level relational and institutional resources, and societal‐level informational factors.

At the individual level, the existing literature indicates that pro‐environmental values function as guiding principles that orient attention toward environmental consequences and increase receptiveness to collective environmental action (Brambati et al. 2022; Soeiro and Ferreira Dias 2020b; Bouman et al. 2018; Steg et al. 2014). More specifically, common good orientation and prosocial values motivate participation in initiatives designed to benefit the broader community (Schwartz 1992). This orientation aligns with RECs' fundamental nature as collective goods that generate shared environmental and social benefits alongside individual advantages. Citizens strongly oriented toward the common good may view REC participation as an opportunity to contribute to community well‐being (Becker and Kunze 2014).

As for civic engagement, there is evidence that those with established patterns of civic participation possess not only relevant skills and networks but also a civic identity that predisposes them toward collective problem‐solving (Dóci and Vasileiadou 2015). This civic orientation facilitates the transition from passive energy consumer to active REC member.

Besides these general orientations, specific motivations toward RECs encompass both intrinsic and extrinsic dimensions (Deci and Ryan 2000). Intrinsic motivation captures citizens' inherent interest in and satisfaction derived from participating in community energy. Extrinsic motivation reflects instrumental considerations, particularly economic benefits such as reduced energy costs or financial returns. Intrinsically motivated participants potentially show greater resilience to challenges and stronger commitment to shared goals (Horstink et al. 2020; Lupi et al. 2021).

REC acceptability—that is, citizens' general evaluation of RECs as positive and beneficial for their territory—also serves as a proximal precursor to participation (Menegatto et al. 2025; Perlaviciute et al. 2017), linking abstract environmental concern and concrete willingness to engage for the ecological transition.

At the community level, sense of community creates the relational foundation necessary for collaborative energy projects (McMillan and Chavis 1986). Communities characterized by strong social bonds and cohesion show greater capacity to organize collective initiatives, mobilize resources, and sustain cooperation over time (Dóci and Vasileiadou 2015; Walker et al. 2010). In addition, trust in local institutions (including municipalities, local governments, and community organizations) proves particularly important for RECs given the regulatory complexities, financial commitments, and long‐term cooperation involved (Devine‐Wright 2011); without confidence in institutional support and governance, citizens may hesitate to commit resources and energy to community projects (Bomberg and McEwen 2012; Hasanov and Zuidema 2022).

Common good orientation reflects individuals' commitment to collective welfare and community benefit (Castiglioni et al. 2019). This orientation aligns naturally with REC's emphasis on shared benefits and collective resource management. Sense of Responsible Togetherness (SORT) captures perceptions of collective responsibility and cooperative dynamics within the community (Procentese et al. 2019; Procentese and Gatti 2019). It reflects the extent to which community members perceive mutual support and shared accountability for collective outcomes, which may facilitate sustained collaboration in REC initiatives.

Subjective norms—perceived expectations from significant others (including friends, family members, and fellow community residents) regarding REC participation (i.e., subjective norms) (Ajzen 1991)—also operate at the community level. These normative perceptions create social facilitation and reduce uncertainty about whether engagement represents an appropriate course of action. When citizens perceive that important others within their local community support or expect their participation, they are more likely to translate personal interest into actual engagement (Conradie et al. 2021).

At the societal level, communication clarity emerged as a frequently cited important factor that can either hinder or promote participation: complex regulatory frameworks, technical terminology, and unclear information about how to join or what membership entails create cognitive obstacles that affect citizens with limited prior knowledge of energy systems (Cabarcos et al. 2020; Spasova and Braungardt 2021). Conversely, clear, transparent, and accessible communication reduces entry barriers and sustains informed decision‐making (Cabarcos et al. 2020; Spasova and Braungardt 2021), specifically when two‐way communication (Standal et al. 2023) and customized messaging (Bauwens 2019) are implemented.

### 2.1 The Present Study

#### 2.1.1 Study Aims and Research Questions

Building on this multilevel framework, the present study employs Latent Profile Analysis (LPA) to identify distinct psychosocial profiles of potential REC members that reflect the interrelationships among the different variables examined in the literature. By employing LPA, the study intended to address the heterogeneity of respondents' profiles, which will potentially allow either higher or lower willingness to participate in REC projects, beyond a mere yes versus no logic. Precisely, the study goal was to understand which profiles were associated with stronger participation intentions. In addition, this study aims to examine how the various profiles emerging from the LPA relate to the different modes of RECs engagement, namely consumer, prosumer, and prosumer‐volunteer.

Employing LPA to investigate citizens' willingness to participate in RECs initiatives is particularly suitable to deeply understand the heterogeneity of participation in RECs. First, understanding profile differences allows us to identify which groups may need targeted support to overcome barriers and which might serve as “bridging actors” to build broader community involvement. Second, emerging profiles illuminate how different participatory pathways contribute to community development and how diverse citizen configurations can create tensions or synergies within RECs. Third, a detailed profile characterization enables the design of tailored communication strategies that focus on specific concerns, values, and capacities of different community subgroups. Fourth, identifying distinct profiles advances theoretical understanding of how multilevel factors combine to shape participation in shared projects for sustainability.

Two primary research questions were at the core of our study.

***Profile Identification*.** How many psychosocial profiles can be identified among potential REC participants, and what characteristics define them?

***Profile Differences in Participation Intentions*.** How do profiles differ in their levels of willingness to participate in RECs and in their preferred modes of participation? Three engagement levels were assessed—consumer only, prosumer, and prosumer with volunteer activities—ordered by increasing commitment and resource investment required.

Although the literature provides evidence on each variable included in this study as either a resource for or an obstacle to participation, to the best of our knowledge, no research has yet applied LPA to examine the combined pattern of factors underlying willingness to participate in RECs projects. Given the exploratory nature of this approach, we did not formulate specific hypotheses about the latent profiles of our participants. Instead, we adopted a bottom‐up approach, focusing on categories that emerged from the data.

### 2.2 The Context of the Study

Italy transposed the European Union's Renewable Energy Directive (RED II) through Legislative Decree 199/2021, establishing the legal framework for RECs. This legislation, which came into force on December 15, 2021, defines RECs as voluntary, open‐participation entities prioritizing community environmental, economic, and social benefits over financial profit, with members geographically connected to the same primary substation (Brunoro et al. 2024; Zhu et al. 2025). The decree significantly expanded participation possibilities compared to the earlier transitional framework (Decree‐Law 162/2019), increasing the power capacity limit from 200 kW to 1 MW and extending membership eligibility from secondary to primary substations.

Italian legislation distinguishes between consumers, who join RECs to consume shared renewable energy, and prosumers, who both produce and consume renewable energy through their own installations. However, research evidence indicates that successful RECs often depend on members who volunteer their time for governance, management, and coordination activities (De Simone et al. 2025). This suggests a third engagement level: prosumer with volunteer activities, combining energy production with active organizational participation. These three modalities—consumer, prosumer, and prosumer‐volunteer—represent increasing levels of commitment in terms of financial investment, time, and civic involvement.

Despite enabling legislation, REC development in Italy remains nascent. Unlike Germany, Denmark, and the Netherlands—where energy cooperatives have evolved over decades with strong institutional support and embedded social practices (Hartmann and Palm 2023; Islar and Busch 2016)—Italy lacks this historical foundation. As of early 2024, approximately 154 operational configurations of energy sharing existed (Eroe et al. 2024), though this number increased to 578 by March 2025 (Clò 2025). However, the total installed capacity remains modest at approximately 50 MW, with most RECs being small‐scale initiatives averaging 8.2 members (Clò 2025). Key barriers to expansion include administrative complexity, regulatory uncertainty, limited public awareness, and difficulties navigating technical requirements (Eroe et al. 2024). The delayed publication of implementation decrees—806 days versus the initially anticipated 180—further constrained development, potentially preventing an estimated 400 additional communities from forming (Eroe et al. 2024).

This nascent developmental stage creates a valuable research opportunity. With limited direct REC experience among Italian citizens at the time of data collection (March–April 2024), identifying psychosocial profiles associated with participation willingness can inform targeted engagement strategies during this critical formative period. Understanding which citizen psychosocial configurations facilitate REC adoption may accelerate Italy's energy transition—which targets 55.4% of gross final electricity consumption from renewables by 2030 (Zhu et al. 2025)—while promoting broader social inclusion in the energy sector.

## 3 Materials and Methods

### 3.1 Participants and Procedure

Data were collected between March and April 2024 through a professional panel provider, with a screening question ensuring that only individuals not currently being members of RECs were included in the sample. An online questionnaire was implemented on the Qualtrics platform, with a stratified quota sampling approach to ensure adequate representation by age and gender within each community. The study was approved by the Ethical Committee of Psychological Research of the Department of Humanities of the University of Naples Federico II with protocol number 1/2025 (date of approval: January 15, 2025) and conducted in accordance with the Declaration of Helsinki. All participants provided informed consent before participation. The average survey completion time was approximately 30 min.

Respondents were 900 Italian citizens (51.4% female) aged between 18 and 84 (*M* = 50.64; SD = 14.33). They were from 30 different municipalities across Italy. Communities were distributed across geographic areas as follows: North (*n* = 300, 33.3%), Center (*n* = 300, 33.3%), and South and Islands (*n* = 300, 33.3%).

Data for this study were collected as part of a larger research project examining REC participation in Italy. The same dataset has been used to address different research questions through complementary analytical approaches: the present study employs LPA to identify psychosocial profiles, while a separate manuscript (currently under review) examines multilevel predictors of participation intentions using hierarchical linear modeling. These two approaches are conceptually distinct and non‐overlapping: LPA is a person‐centered technique that informs *who* the subgroups are, whereas HLM is a variable‐centered technique that informs *which* predictors matter on average across the population. The two manuscripts therefore address different research questions, report different analyses, and yield complementary rather than redundant findings.

### 3.2 Sample Selection

Prior to analysis, data were screened for multivariate outliers using Mahalanobis distance (*χ* ^2^ criterion, *p* < 0.001), resulting in the exclusion of five cases. Of the remaining 895 participants, 314 (35.1%) had missing data on perceived communication clarity. This missingness was by design: the survey included a filter question asking whether participants had ever heard about RECs, and only those who responded affirmatively were subsequently asked to evaluate the clarity of REC‐related information. We adopted a complete case analysis approach (*N* = 580) to avoid potentially biased imputation. Comprehensive missing data analysis, including comparisons between completers and non‐completers and sensitivity analyses testing demographic predictors of profile membership, is provided in Supporting Information S1: Appendix A.

### 3.3 Measures

All measures used 5‐point Likert scales unless otherwise specified. Reliability indices for all scales are presented in Table 1.

**Table 1:** Descriptive statistics for profile indicators and outcome variables.

Variable	*M*	SD	Min	Max	*α*	*ω*
REC acceptability	4.91	0.94	1.86	7.00	0.73	0.73
Pro‐environmental values	5.70	1.18	1.00	7.00	0.92	0.92
Civic engagement	3.82	1.48	1.00	7.00	0.91	0.92
Intrinsic motivation	3.72	0.93	1.00	5.00	0.87	0.87
Extrinsic motivation	3.52	0.84	1.00	5.00	0.65	0.73
Sense of community	3.12	0.87	1.00	5.00	0.91	0.91
Local institutional trust	3.98	1.49	1.00	7.00	0.95	0.95
Common good orientation	6.30	1.55	1.00	9.00	0.88	0.89
SORT	2.83	0.49	1.00	4.00	0.95	0.95
Subjective norms	3.29	0.95	1.00	5.00	0.90	0.90
Communication clarity	2.89	1.01	1.00	5.00	0.90	0.90
Intention to participate	0.64	0.48	0	1	—	—
Engagement level^a^	1.63	0.73	1	3	—	—

*Note: N* = 580.

^a^ Among participants willing to participate only (*n* = 369). Intention to participate coded as 0 = no, 1 = yes. Engagement level coded as 1 = consumer only, 2 = prosumer, 3 = prosumer with volunteer activities.

### 3.4 Profile Indicators

Eleven variables were selected as profile indicators based on their relevance to REC participation.

*REC Acceptability* was assessed using three items adapted from the Public Acceptability of Energy Projects scale (Perlaviciute et al. 2017). The scale measures perceived impact and general acceptability of RECs. Items were rated on a 7‐point scale (1 = strongly disagree; 7 = strongly agree). Example item: “Renewable energy communities will contribute to climate change mitigation.”

*Pro‐environmental Values* were measured using four items from the Environmental Portrait Values Questionnaire (E‐PVQ; Bouman et al. 2018; adapted from Steg et al. 2014). The scale captures the importance individuals attribute to environmental protection and nature conservation. Items were rated on a 7‐point scale (1 = not at all like me; 7 = very much like me). Example item: “Respecting nature is important to this person.”

*Civic Engagement* was assessed using 14 items from the Civic Engagement Scale (Doolittle and Faul 2013), items were rated on a 7‐point scale (1 = strongly disagree; 7 = strongly agree). The scale captures participants' involvement in community activities and their sense of civic responsibility. Example item: “I feel responsible for my community.”

*Extrinsic/Intrinsic Motivation toward RECs* (adapted from Tabernero and Hernández 2011) was assessed using six items rated on a 5‐point scale (1 = not at all; 5 = completely). Participants were asked: “To understand the reasons that motivate participation or interest in renewable energy communities, please indicate the main reasons that drive or would drive you to participate in a REC.” The scale included three extrinsic motivation items (e.g., “Because I have the possibility of saving money on my energy bill”) and three intrinsic motivation items (e.g., “Because I have the possibility of contributing to something important for the environment”).

*Sense of Community (SOC)* was measured using the eight‐item Brief Sense of Community Scale (BSCS; Peterson et al. 2008). Items were rated on a 5‐point scale (1 = strongly disagree; 5 = strongly agree). The scale assesses feelings of membership, mutual influence, fulfillment of needs, and shared emotional connection within one's territorial community. Example item: “I feel like a member of my neighborhood/community.”

*Trust in Local Institutions* was assessed using five items adapted from the Competence‐based and Integrity‐based Trust Scale (Palomo‐Vélez et al. 2024). Items assessed trust in the local municipality's competence (e.g., “Has the necessary knowledge”) and integrity (e.g., “Is transparent in its communication”) regarding REC initiatives, on a 7‐point scale from (1 = not at all; 7 = very much).

*Common Good Orientation* was measured using seven items from the Common Good Provision (CGP) scale (Castiglioni et al. 2019). Items were rated on a 9‐point scale (1 = not at all like me; 9 = very much like me). Participants were asked to indicate their level of agreement with reasons for contributing to the common good, introduced by the stem: “If I contribute to the common good, I do it mainly to….” Example item: “…contribute to meeting everyone's needs.”

*Sense of Responsible Togetherness (SORT)* was assessed using 25 items from the SORT scale (Procentese et al. 2019; Procentese and Gatti 2019). Items were rated on a 5‐point scale (1 = strongly disagree; 5 = strongly agree). The scale captures perceptions of collective responsibility and cooperative dynamics within the community. Example item: “In my community, people help each other in daily neighborhood activities.”

*Subjective Norms* were measured using four items from the Subjective Norms Scale (Conradie et al. 2021), assessing perceived social pressure and support from family, friends, and community members regarding REC participation. Items were rated on 5‐point scale (1 = strongly disagree; 5 = strongly agree). Example item: “My family would approve my participation in a renewable energy community.”

*Perceived REC Communication Clarity* was assessed using three items developed ad hoc based on constructs emerging from the systematic review (De Simone et al. 2025) and media analysis (D'Errico et al. 2025; Scardigno et al. 2025) conducted in earlier phases of the project. Items were rated on 5‐point scale (1 = not clear at all; 5 = completely clear). The scale measures perceived clarity and accessibility of information about REC engineering, legal, and economic issues. Example item: “How clear were the following aspects for you?.”

### 3.5 Outcome Variables

To establish the practical relevance and external validity of the identified profiles, two outcome variables were examined. These variables were not included in the profile estimation, allowing us to test whether profile membership predicts actual participation intentions and preferences.

*Intention to participate* in RECs was assessed with a single dichotomous ad hoc item asking participants whether they would join a REC project in their municipality (0 = no, 1 = yes). Among participants who indicated their willingness to participate (*n* = 560, 62.6%), their preferred *engagement level* was assessed with a categorical item asking them how they would have liked to participate in a REC, offering three options: consumer only (*n* = 290, 51.8%), prosumer (*n* = 183, 32.7%), or prosumer with volunteer activities (*n* = 87, 15.5%).

### 3.6 Sociodemographic Variables

Participants reported their age, gender, education, and municipality of residence. Age was measured in years. Gender was coded as male/female. Education was assessed using six categories ranging from elementary school to post‐graduate degree. Municipalities were aggregated into three macro‐regions (North, Center, and South‐Islands) for the analyses. These variables were used for profile characterization and validation analyses.

### 3.7 Data Analyses

Our analytical approach combined LPA with multivariate analysis of variance (ANOVA) to provide a comprehensive understanding of participation patterns in RECs projects. First, we employed LPA, a mixture modeling technique particularly well‐suited to identifying distinct subpopulations characterized by different configurations of continuous variables (Spurk et al. 2020). Unlike variable‐centered approaches that examine how individual factors independently predict outcomes, LPA identifies patterns in how multiple characteristics combine within individuals, revealing qualitatively distinct profiles that may require different engagement approaches (Howard and Hoffman 2018; Morin and Marsh 2015). This person‐centered technique allowed us to uncover naturally occurring subgroups within our sample based on participants' scores across the variables included in the study. Second, once the latent profiles were identified, we conducted a chi‐square test of independence to examine the associations between profile membership and key outcome variables to determine whether the identified profiles differed significantly in terms of willingness to participate in RECs, as well as to explore potential differences in modes of participation across profiles.

### 3.8 Latent Profile Analysis

LPA was conducted using R version 4.3.1 and the tidyLPA package version 1.1.0 (Rosenberg et al. 2018). Following best‐practice recommendations (Spurk et al. 2020; Weller et al. 2020), we estimated models ranging from 1 to 6 profiles to determine the optimal number of latent profiles. We used Model 1 specification in LPA, which constrains variances to be equal across profiles and covariances among indicators to zero. This model specification balances parsimony with flexibility and is appropriate when profile indicators represent conceptually distinct constructs measured on different scales (Spurk et al. 2020). Models were estimated using the expectation‐maximization (EM) algorithm.

### 3.9 Model Selection Criteria

The optimal number of profiles was determined using multiple statistical indices in combination with theoretical interpretability (Nylund et al. 2007; Spurk et al. 2020). Statistical fit indices included: (a) information criteria (Akaike Information Criterion [AIC], Bayesian Information Criterion [BIC], and sample‐size adjusted BIC [SABIC]), with lower values indicating better fit; (b) likelihood ratio tests comparing *k* and *k*−1 profile models (Lo‐Mendell–Rubin Likelihood Ratio Test [LMR‐LRT] and Bootstrap Likelihood Ratio Test [BLRT]), with significant *p*‐values favoring the *k*‐profile solution; and (c) entropy, indicating classification quality (values > 0.80 considered good). Additionally, we considered practical criteria including the relative size of the smallest profile (> 5% of sample), average posterior probabilities for profile membership (> 0.80), and theoretical meaningfulness and interpretability of the profile solution. Following recommendations to prioritize theoretical interpretability when statistical indices provide mixed signals (Spurk et al. 2020), final model selection balanced statistical fit with substantive meaningfulness (see Supporting Information S1: Appendix B for model fit comparison).

### 3.10 Profile Characterization and External Validation

Once the optimal profile solution was selected, we characterized each profile by examining means and standard errors of all profile indicators. Profile indicators were standardized (*z*‐scores) for visualization and interpretation purposes. Profiles were labeled based on their distinctive patterns across indicators, considering both level differences (overall high vs. low scores) and shape differences (distinctive patterns of highs and lows across specific indicators; Morin et al. 2016). To examine the external validity of the profile solution, we first examined demographic differences across profiles using chi‐square tests (for categorical demographics) and one‐way ANOVAs with Bonferroni post‐hoc tests (for continuous demographics). We then tested whether profiles differed on outcome variables not included in the profile estimation. For the dichotomous intention to participate variable, we conducted chi‐square tests of independence. For the categorical engagement level variable (among those intending to participate), we examined the distribution across profiles using crosstabulation and chi‐square tests. Profile comparisons on outcome variables were conducted using the BCH method (Bakk and Vermunt 2016; Bolck et al. 2004) implemented through the manual three‐step approach, which accounts for classification uncertainty in profile membership while testing associations with distal outcomes. This approach first estimates the latent profile model, then saves classification probabilities, and finally tests differences on external variables while adjusting for measurement error in profile assignment.

### 3.11 Statistical Significance

All statistical tests were evaluated at *α* = 0.05. Given the exploratory nature of the profile analysis and the multiple comparisons involved in profile characterization and validation, we report both statistical significance and effect sizes (Cramér's *V* for categorical associations, Cohen's *d* or *η* ^2^ for continuous group differences) to facilitate interpretation of practical significance.

## 4 Results

### 4.1 Preliminary Analyses

After removing five multivariate outliers, all measurement scales demonstrated adequate to excellent internal consistency reliability (see Table 1 for detailed reliability statistics as reported in the Measures section). Profile indicators showed adequate variability for profile differentiation.

To assess the discriminant validity and independence of profile indicators, we examined bivariate correlations among all variables (Table 2). Correlations ranged from *r* = 0.26 (SORT‐Extrinsic motivation) to *r* = 0.58 (SOC‐SORT), indicating theoretically meaningful associations while maintaining sufficient independence for LPA. The strongest correlations were observed among community‐level variables (e.g., SOC and SORT, *r* = 0.58; SOC and local institutional trust, *r* = 0.53), supporting the theoretical multilevel structure. All indicators showed positive intercorrelations, consistent with a general civic‐environmental engagement dimension, while maintaining adequate discriminant validity for profile differentiation.

**Table 2:** Bivariate correlations among profile indicators.

	1	2	3	4	5	6	7	8	9	10	11
1. REC Acceptability	—										
2. Pro‐environmental values	0.52***	—									
3. Civic engagement	0.45***	0.48***	—								
4. Intrinsic motivation	0.58***	0.55***	0.51***	—							
5. Extrinsic motivation	0.47***	0.38***	0.32***	0.49***	—						
6. Sense of community	0.39***	0.35***	0.48***	0.42***	0.31***	—					
7. Local institutional trust	0.41***	0.34***	0.43***	0.39***	0.28***	0.53***	—				
8. Common good orientation	0.44***	0.49***	0.38***	0.48***	0.35***	0.36***	0.33***	—			
9. SORT	0.38***	0.33***	0.41***	0.37***	0.26***	0.58***	0.54***	0.35***	—		
10. Subjective norms	0.51***	0.46***	0.43***	0.52***	0.41***	0.42***	0.39***	0.45***	0.40***	—	
11. Communication clarity	0.35***	0.28***	0.31***	0.33***	0.28***	0.32***	0.38***	0.29***	0.34***	0.35***	—

*Note: N* = 580. All correlations are based on standardized scores.

*** *p* < 0.001.

### 4.2 Latent Profile Enumeration

Following best‐practice recommendations (Spurk et al. 2020), we systematically compared latent profile models ranging from 1 to 6 profiles (Table 3).

**Table 3:** Model fit indices for 1–6 profile solutions.

Profiles	AIC	BIC	Entropy	n_min	n_max	BLRT_p
1	16,183.72	16,275.78	—	580	580	—
2	15,979.50	16,140.16	0.873	171	409	< 0.001
**3**	**15,823.02**	**16,052.28**	**0.897**	**47**	**327**	**< 0.001**
4	15,772.72	16,070.58	0.886	38	254	< 0.001
5	15,763.86	16,130.32	0.885	36	223	0.088
6	15,755.52	16,190.58	0.900	26	193	0.360

*Note: N* = 580. Bold indicates the selected model.

Abbreviations: AIC, Akaike Information Criterion; BIC, Bayesian Information Criterion; BLRT_p, Bootstrap Likelihood Ratio Test *p*‐value; n_min/n_max, smallest/largest profile size.

Based on the comprehensive evaluation of fit indices, we selected the three‐profile solution as optimal. While AIC continued to decrease slightly through six profiles, the three‐profile solution provided (a) the best balance of parsimony (lowest BIC among substantively interpretable solutions), (b) excellent classification quality (entropy = 0.897, substantially above the 0.80 threshold), (c) adequate minimum profile size (*n* = 47, 8.1% of sample, exceeding the 5% threshold), (d) significant improvement over simpler models (BLRT *p *< 0.001), and (e) theoretically meaningful and distinct profiles aligned with our multilevel framework. The 4–6 profile solutions, while showing marginally better AIC values, produced increasingly smaller profiles with diminishing theoretical distinctiveness and non‐significant BLRT values for 5–6 profiles.

### 4.3 Profile Characterization

**Figure 1:**
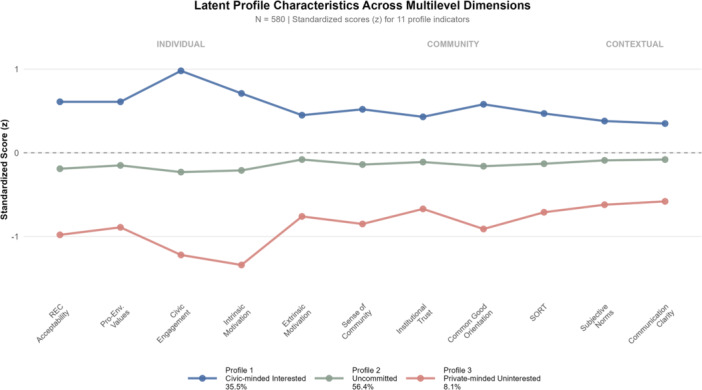
Latent profile analysis: Standardized scores across psychosocial dimensions.

The three‐profile solution revealed distinct psychosocial patterns across the 11 indicators spanning across individual, community, and societal levels (Table 4, Figure 1). One‐way ANOVAs confirmed significant mean differences across profiles for all indicators (all *p*s < 0.001, *η* ^2^s ranging from 0.08 to 0.26). Post hoc pairwise comparisons (Tukey HSD) indicated that all three profiles differed significantly from each other on most indicators, with few exceptions where Profile 2 overlapped with either Profile 1 or Profile 3. Figure 1 illustrates the distinctive configuration of each profile across the multilevel framework.

**Table 4:** Latent profile means (standard errors) for standardized indicators.

Level/Indicator	Profile 1: Civic‐minded Interested (*n* = 206, 35.5%)	Profile 2: Uncommitted (*n* = 327, 56.4%)	Profile 3: Private‐minded Uninterested (*n* = 47, 8.1%)	*F*	*η* ^2^
Individual level					
Acceptability	0.52 (0.07)^a^	0.01 (0.05)^b^	−0.89 (0.11)^c^	67.4***	0.13
Pro‐environmental values	0.61 (0.07)^a^	−0.08 (0.05)^b^	−1.05 (0.13)^c^	89.2***	0.17
Civic engagement	0.98 (0.06)^a^	−0.31 (0.04)^b^	−1.22 (0.13)^c^	156.8***	0.26
Intrinsic motivation	0.71 (0.06)^a^	−0.12 (0.04)^b^	−1.34 (0.12)^c^	134.7***	0.23
Extrinsic motivation	0.45 (0.07)^a^	−0.02 (0.04)^b^	−0.89 (0.13)^c^	52.3***	0.10
Community level					
Sense of community	0.47 (0.07)^a^	−0.05 (0.04)^b^	−0.92 (0.12)^c^	58.9***	0.12
Local institutional trust	0.52 (0.07)^a^	−0.12 (0.04)^b^	−0.78 (0.12)^c^	74.2***	0.14
Common good orientation	0.34 (0.07)^a^	0.05 (0.04)^b^	−0.89 (0.13)^c^	39.8***	0.08
SORT	0.45 (0.07)^a^	−0.04 (0.04)^b^	−0.84 (0.12)^c^	53.7***	0.11
Subjective norms	0.58 (0.06)^a^	−0.14 (0.04)^b^	−0.89 (0.12)^c^	81.4***	0.15
Societal level					
Communication clarity	0.23 (0.07)^a^	−0.01 (0.04)^b^	−0.48 (0.12)^c^	14.8***	0.03

*Note: N* = 580. Values are standardized mean scores (*z*‐scores) with standard errors in parentheses. Superscript letters (^a, b, c^) indicate results of Tukey HSD post hoc tests: means sharing the same superscript letter do not differ significantly at *p* < 0.05; means with different superscripts differ significantly from each other. All one‐way ANOVAs testing differences across the three profiles were significant at

*** *p* < 0.001. *η* ^2 ^= partial eta‐squared effect size, interpreted as 0.01 = small effect, 0.06 = medium effect, 0.14 = large effect (Cohen 2013).

The three profiles that emerged are:

**Profile 1: Civic‐minded Interested (** ***n*** = **206, 35.5%).** Overall, this profile comprises individuals who are civically active, environmentally aware, socially integrated, confident in local institutions, and perceive strong normative support for REC participation. Unlike the other profiles, this one includes citizens who exhibited consistently elevated scores across all indicators, particularly on civic engagement (+0.98 SD), pro‐environmental values (+0.61 SD), and intrinsic motivation (+ 0.71 SD). Participants in this profile also showed levels of community indicators substantially above the average: local institutional trust (+0.52 SD), SOC (+0.47 SD), and SORT (+0.45 SD), as well as societal indicators such as subjective norms (+0.58 SD). Extrinsic motivation (+0.45 SD) and common good orientation (+0.34 SD) were moderately elevated. Only communication clarity was slightly above the average (+0.23 SD).

**Profile 2: Uncommitted (** ***n*** = **327, 56.4%).** This is the largest profile, capturing a “psychosocially moderate” majority who lacks strong orientations toward or against RECs. Citizens belonging to this profile exhibited slight negative deviation levels on all the indicators except for Common good orientation, which was slightly positive (+0.05 SD). As for the other variables, people in this subgroup reported almost average levels on civic engagement (−0.31 SD), subjective norms (−0.14 SD), intrinsic motivation (−0.12 SD), and local institutional trust (−0.12 SD). Pro‐environmental values (−0.08 SD), SOC (−0.05 SD), SORT (−0.04 SD), extrinsic motivation (−0.02 SD), communication clarity (−0.01 SD), and acceptability (+0.01 SD) hovered around average.

**Profile 3: Private‐minded Uninterested (** ***n*** = **47, 8.1%).** This profile included individuals who are civically disengaged, lack environmental concern, perceive limited normative support for REC engagement, and hold less favorable views of community institutions. These people exhibited substantially below‐average scores across all indicators, most notably on intrinsic motivation (−1.34 SD), civic engagement (−1.22 SD), and pro‐environmental values (−1.05 SD). Moreover, they also showed substantially lower REC acceptability (−0.89 SD), extrinsic motivation (−0.89 SD), subjective norms (−0.89 SD), common good orientation (−0.89 SD), SOC (−0.92 SD), SORT (−0.84 SD), local institutional trust (−0.78 SD), and communication clarity (−0.48 SD).

Profile membership showed no significant associations with age, *F*(2, 577) = 0.38, *p* = 0.684, gender, *χ* ^2^(2) = 3.36, *p* = 0.186, education, *χ* ^2^(8) = 5.18, *p* = 0.738, or geographic region, *χ* ^2^(4) = 1.20, *p* = 0.878.

### 4.4 Profile Differences in REC Participation Outcomes

To examine whether the identified psychosocial profiles have practical implications for REC participation, we tested associations between profile membership and two outcome variables: intention to participate and preferred participation modality. Critically, these outcome variables were not used in the profile identification process, allowing us to assess whether the profiles predict meaningful differences in participation patterns beyond the indicators used to define them.

#### 4.4.1 Primary Outcome: Intention to Participate

In the total sample (*N* = 580), 63.6% of our respondents (*n* = 369) indicated that they were willing to join a hypothetical REC in their municipality, while 36.4% (*n* = 211) indicated they were not.

Although chi‐square analysis did not reveal a statistically significant association between profile membership and intention to participate, *χ* ^2^(2) = 4.42, *p* = 0.110, Cramér's *V* = 0.09 (Table 5), examination of the distribution pattern suggests complexity worth noting.

**Table 5:** Intention to participate by latent profile.

Profile	Willing to participate	Not willing to participate	Total
Civic‐minded Interested	138 (67.0%)	68 (33.0%)	206
Uncommitted	197 (60.2%)	130 (39.8%)	327
Private‐minded Uninterested	34 (72.3%)	13 (27.7%)	47
Total	369 (63.6%)	211 (36.4%)	580

*Note: χ* ^2^(2) = 4.42, *p* = 0.110, Cramér's *V* = 0.09. Percentages calculated within each profile.

Profile 3 (Private‐minded Uninterested) showed the highest proportion of participants willing to participate (72.3%), followed by Profile 1 (Civic‐minded Interested, 67.0%), and Profile 2 (Uncommitted, 60.2%). While these differences must be interpreted with caution given the lack of statistical significance, the unexpected ordering—particularly the high stated willingness among those with the lowest psychosocial engagement—raises questions about the intention‐behavior relationship in REC contexts that merit further investigation with larger samples and behavioral outcomes.

#### 4.4.2 Secondary Outcome: Engagement Level

Among participants who indicated their willingness to join a REC (*n* = 369), 52.3% (*n* = 193) preferred the lowest engagement level (consumer only), 32.5% (*n* = 120) as prosumers, and 15.2% (*n *= 56) as prosumers with volunteer activities (Table 6).

**Table 6:** Engagement level by latent profile among willing participants.

Profile	Consumer only	Prosumer	Prosumer + Volunteer	Total
Civic‐minded Interested	68 (49.3%)	42 (30.4%)	28 (20.3%)	138
Uncommitted	108 (54.8%)	67 (34.0%)	22 (11.2%)	197
Private‐minded Uninterested	17 (50.0%)	11 (32.4%)	6 (17.6%)	34
Total	193 (52.3%)	120 (32.5%)	56 (15.2%)	369

*Note: χ* ^2^(4) = 5.44, *p* = 0.246, Cramér's *V* = 0.07. Percentages calculated within each profile.

Chi‐square analysis revealed no significant association between the profile membership and engagement level, *χ^2^* (4) = 5.44, *p* = 0.246, Cramér's *V* = 0.07. While psychosocial profiles differentiated citizens' overall orientations toward REC, the community of residence, and the environment, they do not determine the intensity of engagement individuals are willing to commit when deciding to become REC members.

## 5 Discussion

Our findings identified three distinct psychosocial profiles among Italian citizens, corroborating that the population of potential REC members is not homogeneous but includes qualitatively different subgroups of persons characterized by distinct combinations of individual, community‐related, and society‐based factors. Critically, however, profile membership was not statistically associated with either intention to participate or preferred engagement level. This null finding is itself the primary result to discuss: the psychosocial configurations that differentiate citizens across 11 indicators do not translate, at least in this sample and with these outcome measures, into meaningfully different rates of stated willingness to join a REC.

A possible explanation for the absence of significant associations lies in the methodological asymmetry between profile indicators, derived from multi‐item Likert scales with good reliability, and outcomes, which relied on a single dichotomous item (intention) and a single categorical item (engagement level). Such a mismatch in psychometric resolution is likely to attenuate any association between profiles and outcomes, regardless of the theoretical validity of the profiles themselves.

Beyond measurement issues, the finding may also reflect a substantive feature of hypothetical participation scenarios: when the decision to join a REC carries no real consequences, stated willingness may function as a general expression of openness rather than a differentiated commitment shaped by specific psychosocial resources. Research on hypothetical bias suggests that costless scenarios tend to inflate stated willingness independently of individual differences in underlying dispositions (Ajzen et al. 2004), which would explain why profiles that differ substantially on civic and motivational resources converge on similar rates of declared intention. Profile differences would thus be expected to emerge more clearly in longitudinal designs linking pre‐participation profiles directly to objective behavioral outcomes—actual enrollment, sustained participation, dropout—rather than to stated intentions alone (Carrington et al. 2014).

With these caveats in mind, we note, purely as a non‐significant descriptive trend requiring replication, that Profile 3 (*Private‐minded Uninterested*) showed numerically higher stated willingness (72.3%) than Profile 1 (*Civic‐minded Interested*, 67.0%) and Profile 2 (*Uncommitted*, 60.2%). Should this pattern prove robust in future studies with differently measured outcome variables, it might suggest that highly engaged citizens respond more cautiously due to a realistic appraisal of what collective action truly demands (Lupi et al. 2021; Young and Brans 2017); while less engaged citizens may overestimate their readiness, as emerged in documented patterns of overconfidence among individuals with limited domain knowledge (Kruger and Dunning 1999).

The collective nature of RECs may further widen the gap between hypothetical willingness and actual engagement. Unlike individual pro‐environmental behavior (e.g., purchasing solar panels for one's home), REC participation requires ongoing coordination with the other REC members, trust in the governance structures, and sustained engagement with collective decision‐making (Gregg et al. 2020; Walker et al. 2010). These collective demands may not be fully appreciated by potential participants at the intention stage, regardless of their psychosocial profile. Supporting this interpretation, our analysis revealed no significant differences across profiles in preferred engagement levels (consumer‐only, prosumer, or prosumer‐with‐volunteer). This uniform distribution suggests that the gap between stated intentions and the realities of REC participation may operate independently of citizens' psychosocial orientations, reflecting instead a more general lack of concrete understanding about what different participation levels entail in practice.

It is worth noticing that perceived communication clarity showed the smallest effect size across the three profiles (*η* ^2^ = 0.03) and that all three profiles reported below‐average communication clarity (ranging from −0.48 to +0.23 SD). This finding indicates that unclear, inaccessible information about RECs represents a systemic barrier shared by all citizens regardless of their motivational orientations. This outcome aligns with existing evidence reporting informational barriers in REC development across European contexts (Spasova and Braungardt 2021). Indeed, it suggests that communication improvements could benefit all citizen segments.

Our analysis revealed that psychosocial profiles were not associated with demographic characteristics (age, gender, education) or geographic region. This finding contrasts with some previous research suggesting that gender may influence participation in renewable energy initiatives (Cabarcos et al. 2020). The absence of demographic effects in our study suggests that psychosocial orientations toward RECs transcend traditional sociodemographic categories, at least in the Italian context at this developmental stage. However, this finding should be interpreted cautiously, as our sample achieved relatively balanced demographic representation through quota sampling, potentially limiting variability in these characteristics compared to more naturalistic recruitment approaches.

In conclusion, our findings indicate that three profiles differ primarily in their overall level of engagement across indicators, representing high, moderate, and low positions along a civic‐ engagement continuum. This level‐dominant structure is consistent with the pattern of bivariate correlations among profile indicators, which were uniformly positive and suggest the presence of a general civic‐environmental engagement dimension underlying all 11 variables. When indicators share a common underlying dimension of this kind, LPA tends to recover level‐based rather than shape‐based profiles, a known feature of the method rather than a limitation specific to this study (Spurk et al. 2020). Importantly, even level‐dominant profiles carry substantive meaning: they identify naturally occurring subgroups that differ in the overall accumulation of psychosocial resources relevant to REC participation, which a single composite index would obscure by collapsing individual variation into a single score that engagement resources tend to accumulate (or remain limited) coherently across individual, community, and societal dimensions rather than showing configurational distinctiveness. The lack of significant association between profiles and participation intentions, combined with this coherent patterning, suggests that stated intentions in hypothetical scenarios may be influenced by factors beyond the psychosocial orientations captured in our profile indicators, reinforcing the need for research linking profiles to actual participation behavior.

## 6 Implications for Intervention, Limitations, and Conclusion

Despite the intention‐behavior puzzle, our profiles have clear implications for strategies aimed at REC development. The *Civic‐minded Interested* profile represents the natural leadership base, possessing civic skills and community networks facilitating collective action. Yet their moderate willingness may reflect the civic overload that results from being aware of the challenges, demands, and constraints of engagement. Participation fatigue—where community members withdraw from projects after being asked repeatedly to contribute across multiple initiatives—is well‐documented in the context of commons management (Poteete et al. 2010; Wilson 2012). REC developers should approach this group with specific proposals aligned with existing civic portfolios, mobilizing them strategically for governance roles rather than generic membership campaigns. Rather than interpreting cautious responses as disinterest, organizers should acknowledge legitimate concerns about coordination challenges, emphasize how RECs differ from other collective action experiences, and position them as “bridging actors” translating REC initiatives into familiar community structures.

The *Uncommitted* profile represents the primary expansion target. Their middling scores suggest latent potential rather than opposition, yet they showed the lowest willingness. Communication should emphasize concrete benefits, transparency, and low‐threshold participation with graduated engagement pathways (Lupi et al. 2021). Providing clear information about participation requirements, addressing specific barriers like economic concerns or unclear information, and using peer testimonials can convert latent interest into participation. Investment in outreach materials, demonstration projects, and consumer‐only membership options, allowing gradual escalation, may prove particularly effective.

Finally, the *Private‐minded Uninterested* participants may join but quickly drop out when participation demands exceed civic capacity (Seyfang et al. 2013). Because this profile tends to underestimate the costs and demands of REC participation, members are at heightened risk of early withdrawal. Rather than assuming that declared intention equals reliable commitment, REC developers should recognize these members' need for structured support in developing the social and organizational competencies required for sustained participation. This includes accessible onboarding, mentorship by experienced members, and explicit community‐building activities aimed at cultivating trust and relational ties.

These profile‐specific recommendations raise an important question for future research: what composition of citizen profiles best supports long‐term REC sustainability? While our cross‐sectional design cannot address this question, existing literature on collective action suggests that successful initiatives typically require diverse forms of contribution (Poteete et al. 2010). Future longitudinal research should examine whether RECs benefit from a mix of highly engaged members providing leadership, moderate members participating when provided with clear structures and accessible entry points, and supported low‐engaged members whose participation is scaffolded through mentorship and community‐building. Understanding optimal profile configurations could inform more strategic approaches to REC member recruitment and retention.

Our cross‐sectional design with hypothetical scenarios cannot establish causality or predict actual behavior. The observed pattern—where citizens with the lowest psychosocial engagement showed numerically higher stated intention to participate in a REC, despite the lack of statistical significance—underscores the need for longitudinal research. This would involve following potential participants from the manifestation of their initial interest to their actual enrollment as members of a REC. Only prospective studies linking pre‐participation profiles with objective behavioral outcomes can determine whether psychosocial profiles truly predict who builds, sustains, and benefits from RECs. Future research should pursue partnerships with operational RECs to link the psychosocial profiles identified through surveys with objective behavioral data from membership records. For example, comparing survey‐based profiles of potential members with administrative data tracking actual enrollment, sustained participation, and dropout patterns would provide crucial external validation of whether psychosocial profiles truly predict participation trajectories.

Our measurement approach may have been insufficiently sensitive, and this limitation is directly relevant to interpreting the null associations between profiles and outcomes. The binary intention measure and the categorical engagement level variable represent single‐item assessments with limited psychometric resolution, whereas profile indicators were derived from validated multi‐item scales. This asymmetry between predictor richness and outcome simplicity likely attenuated any association, and should be addressed in future research through more sensitive outcome measures, including conditional scenarios, implementation intentions that capture specific behavioral plans (Gollwitzer and Sheeran 2006), and graduated commitment scales to distinguish passive interest from concrete planning. Additionally, our reliance on self‐report measures introduces potential biases, including social desirability effects that may have inflated reported pro‐environmental attitudes and participation intentions. The cross‐sectional survey design also prevented us from assessing actual participation behavior, limiting our ability to validate whether stated intentions translate into concrete action. Furthermore, our study did not assess household income or other economic variables that previous research has identified as relevant to renewable energy participation (Cabarcos et al. 2020; Klaniecki et al. 2019). Including economic indicators in future profile analyses could reveal whether financial capacity intersects with psychosocial orientations in shaping participation patterns.

Longitudinal research is needed to determine whether psychosocial profiles remain stable characteristics or are transformed by REC participation itself. Measuring individuals before and after joining a REC would clarify whether engagement experiences enhance civic capacities and community orientations or whether profiles primarily function as selection mechanisms determining who joins and persists.

Our focus on psychosocial factors bracketed structural and economic considerations. Future research should integrate person‐centered profiles with analyses of how economic incentives, regulatory frameworks, and infrastructural constraints interact with psychosocial configurations. The effectiveness of different engagement strategies may vary not only by profile but also by the economic and regulatory context in which RECs operate.

Finally, the exploratory nature of LPA and our specific sampling limit generalizability. While our profile solution demonstrated strong statistical properties and theoretical interpretability aligned with our multilevel framework, replication across diverse geographic, cultural, and policy contexts would be essential. The profiles identified here emerged within the Italian context characterized by nascent REC development and specific regulatory frameworks. Whether similar configurations emerge in contexts with more established REC traditions or different policy environments remains an empirical question.

Despite these limitations, the person‐centered approach employed in this study afforded important theoretical and practical contributions to understanding citizens' intention to participate in RECs. Methodologically, LPA enabled us to move beyond variable‐centered analyses that examine how individual factors independently predict outcomes, revealing instead how multiple psychosocial characteristics combine within individuals to create qualitatively distinct participation pathways (Howard and Hoffman 2018; Morin and Marsh 2015). This analytical shift—from examining isolated variables to identifying naturally occurring configurations of characteristics—remains valuable even when profiles do not significantly predict outcomes in a given sample, establishing the existence and internal coherence of subgroups that research can track longitudinally.

Theoretically, the structure of the profiles is itself a finding with implications for ecological systems theory (Bronfenbrenner 1989). The fact that engagement resources accumulate or remain limited coherently across individual, community, and societal levels simultaneously suggests that these levels do not operate independently but are deeply intertwined within persons. This cross‐level coherence refines ecological systems theory in the REC domain: It indicates that interventions targeting a single level in isolation (e.g., improving communication clarity at the societal level without addressing civic disengagement at the individual level) may be insufficient for citizens whose limited resources span multiple levels simultaneously. Conversely, citizens who are well‐resourced at one level tend to be well‐resourced across all levels, suggesting that engagement may be self‐reinforcing once a threshold of civic‐environmental orientation is reached. These theoretical propositions, while speculative at this stage, represent testable hypotheses for future longitudinal and intervention research. Practically, identifying distinct citizen profiles enables the development of differentiated engagement strategies tailored to specific population segments rather than relying on “one‐size‐fits‐all” approaches. Understanding which psychosocial configurations characterize different potential REC participants allows practitioners to design targeted interventions that address the specific concerns, values, and capacities of different community subgroups, potentially increasing both the breadth and sustainability of citizen engagement in RECs.

## Author Contributions

Evelyn De Simone, Alessia Rochira, and Terri Mannarini developed the primary theoretical framework. Evelyn De Simone performed the latent class analysis and statistical modeling and drafted the first version of the discussion section. Alessia Rochira, Fortuna Procentese, Flora Gatti, Biagio Marano, Francesca D'Errico, Rosa Scardigno, and Carmela Sportelli contributed to refining the theoretical framework and interpreting the results. Terri Mannarini was responsible for project coordination.

## Ethics Statement

This study was approved by the Ethical Committee of Psychological Research of the Department of Humanities of the University of Naples Federico II (approval number: 1/2025; date of approval: January 15, 2025) and was conducted in accordance with the Declaration of Helsinki. All participants provided written informed consent prior to their participation.

## Conflicts of Interest

The authors declare no conflicts of interest.

## Supporting information


Supporting File

## Data Availability

The data that support the findings of this study are available from the corresponding author upon reasonable request.
