# Supplementary material for: Community‐Based Projects for Energy Transition: Citizen Profiling Through Psychological and Social Drivers
Source: J Community Psychol. 2026 Jul 13;54(5):e70131. doi: 10.1002/jcop.70131 (PMC13365645; doi:10.1002/jcop.70131)

**Appendices**

These supplementary materials provide additional detail on: (1) comprehensive missing data analysis justifying complete case approach (Appendix A), and (2) model fit comparison for profile enumeration (Appendix B). All analyses conducted in R version 4.3.1 using tidyLPA package version 1.1.0

**Appendix A
Missing Data Analysis and Sample Selection**

Missing data on communication clarity (35.1%) was not missing completely at random (Little's test *p* < .001). Participants with complete data were slightly older and more likely to be female, with small effect sizes (*d* = 0.13, φ = .07). However, sensitivity analyses revealed that these demographic variables did not predict latent profile membership, suggesting minimal bias in substantive conclusions. Complete case analysis (*N* = 580, 64.9% retention) was adopted as a conservative approach to avoid potentially biased imputation under violated MAR assumptions while maintaining theoretical integrity of the 11-indicator multilevel framework.

**Table S1.** Comparison of Completers vs. Non-Completers

|  | Complete Cases (n = 580) | Missing Cases (n = 314) | Test Statistic | *p*-value | Effect Size |
| --- | --- | --- | --- | --- | --- |
| Age |  |  |  |  |  |
| *M (SD)* | 51.3 (14.5) | 49.4 (13.8) | *t*(668.88) = -2.01 | .045 | *d* = 0.13 |
| Gender (% female) |  |  |  |  |  |
| % | 52.4% | 43.3% | *χ²*(1) = 4.2 | .040 | φ = .07 |
| Education |  |  |  |  |  |
| *M* level | 3.8 | 3.7 | — | .312 | — |
| Region |  |  |  |  |  |
| North | 34.0% | 32.5% | *χ²*(2) = 0.8 | .669 | — |
| Center | 32.8% | 34.1% |  |  |  |
| South-Islands | 33.3% | 33.4% |  |  |  |

*Note.* Completers were slightly older (*d* = 0.13, small effect) and more likely to be female (φ = .07, small effect). No significant differences in education or geographic distribution.

**Do Demographics Predict Profile Membership?**

**Table S2.** Demographic – Profile Membership: Sensitivity analysis

| Predictor | Test | *df* | Statistic | *p*-value | Conclusion |
| --- | --- | --- | --- | --- | --- |
| Age → Profile | ANOVA | 2, 577 | *F* = 0.38 | .684 | No association |
| Gender → Profile | χ² | 2 | 3.36 | .186 | No association |
| Education → Profile | χ² | 8 | 5.18 | .738 | No association |
| Region → Profile | χ² | 4 | 1.20 | .878 | No association |

*Note.* Despite modest demographic differences between completers and non-completers, demographic variables did not predict profile membership among those with complete data. This suggests that selection bias due to missing data is unlikely to substantially affect profile characterization or validation results.

**Appendix B
Model Fit Comparison for 1-6 Profile Solutions**

**Figure S1.** Model Fit Comparison


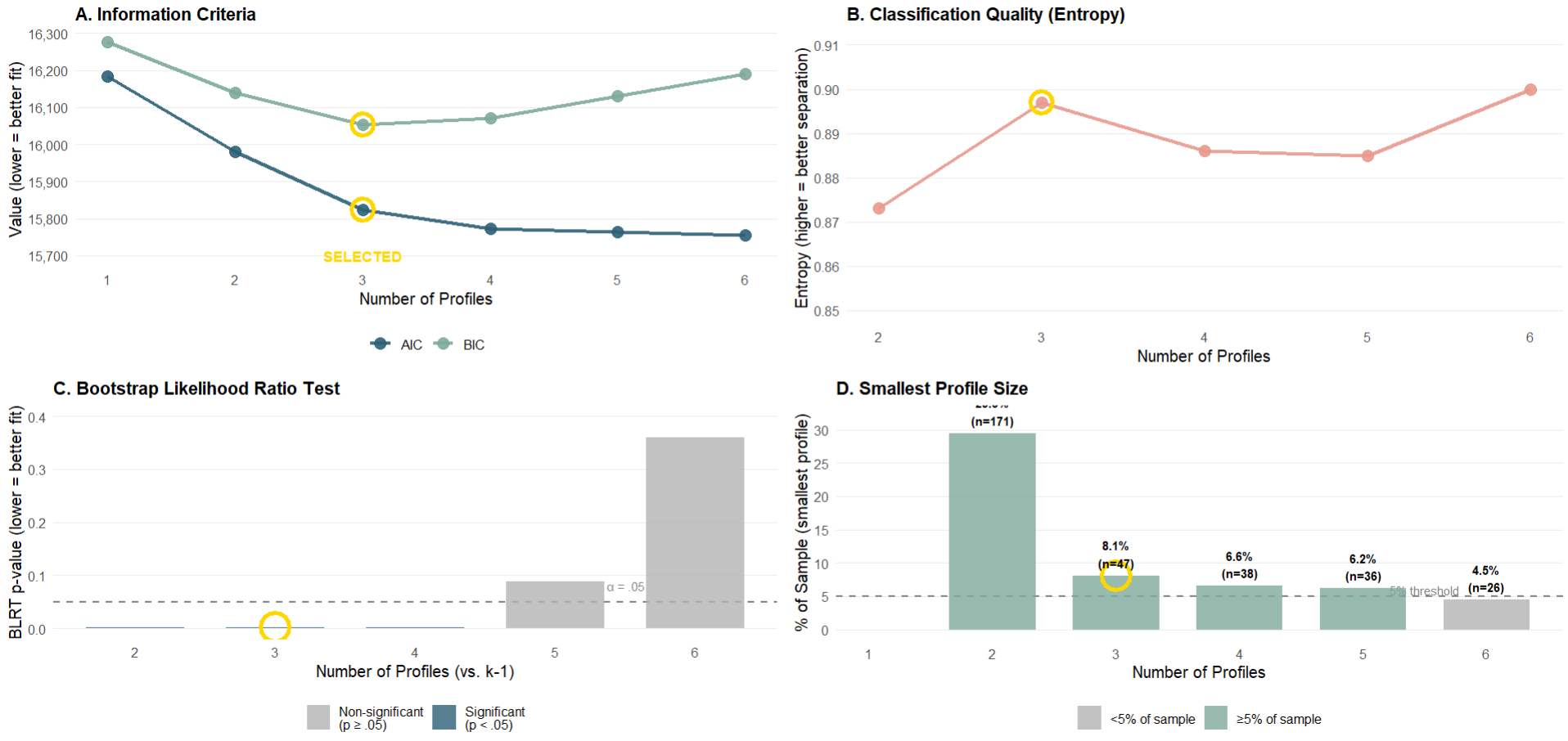

Supplement: Supplementary file 1 — Supporting File [file JCOP-54-0-s001.docx]
